# CD44: molecular interactions, signaling and functions in the nervous system

**DOI:** 10.3389/fncel.2015.00175

**Published:** 2015-05-07

**Authors:** Joanna Dzwonek, Grzegorz M. Wilczynski

**Affiliations:** Laboratory of Molecular and Systemic Neuromorphology, Nencki Institute of Experimental BiologyWarsaw, Poland

**Keywords:** CD44, adhesion molecule, hyaluronan receptor, extracellular matrix receptor

## Abstract

CD44 is the major surface hyaluronan (HA) receptor implicated in intercellular and cell-matrix adhesion, cell migration and signaling. It is a transmembrane, highly glycosylated protein with several isoforms resulting from alternative gene splicing. The CD44 molecule consists of several domains serving different functions: the N-terminal extracellular domain, the stem region, the transmembrane domain and the C-terminal tail. In the nervous system, CD44 expression occurs in both glial and neuronal cells. The role of CD44 in the physiology and pathology of the nervous system is not entirely understood, however, there exists evidence suggesting it might be involved in the axon guidance, cytoplasmic Ca^2+^ clearance, dendritic arborization, synaptic transmission, epileptogenesis, oligodendrocyte and astrocyte differentiation, post-traumatic brain repair and brain tumour development.

## Introduction

CD44 is a transmembrane glycoprotein mediating cell responses to the extracellular microenvironment. Also known as the hermes antigen, Pgp1, MDU3 and Inlu-related p80 glycoprotein, it was first described as a surface molecule present in lymphocytes, thymocytes and granulocytes (Dalchau et al., 1980), and later recognized as a novel human erythrocyte cell surface antigen, lymphocyte homing receptor, and leukocyte surface glycoprotein (Stefanová et al., 1989). Today, CD44 is being described as an adhesion molecule expressed in various cell types, and an important participant in a number of signaling pathways. The present review summarizes the current knowledge on CD44 expression and function in the nervous system.

## Structure

**Figure 1:**
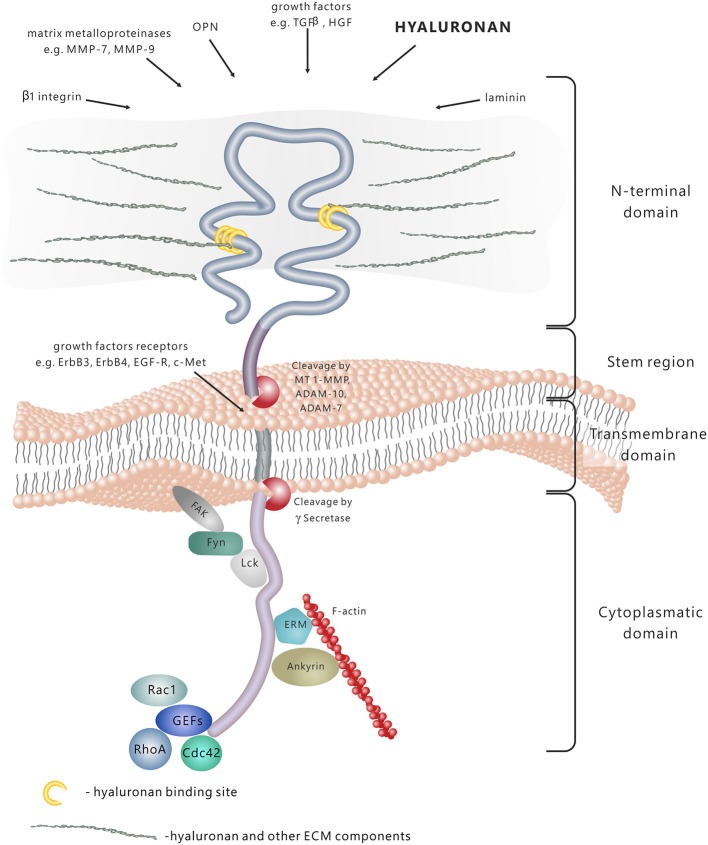
**CD44 protein structure and signaling**. CD44 is a transmembrane molecule composed of several domains. The N-terminal extracellular domain can bind various ligands, including hyaluronan (HA), extracellular matrix (ECM) glycoproteins and proteoglycans, growth factors, cytokines and matrix metalloproteinases. In result of the proteolytic cleavage within the stem region, the extracellular domain is released into the extracellular space. The transmembrane domain anchors and stabilises the molecule in the plasma membrane. The cytoplasmic domain is responsible for signal transduction through binding to different molecules, including cytoskeleton components, kinases and activators of small Rho GTPases (GEFs-guanine nucleotide exchange factors).

CD44 molecule consists of several functional domains. The N-terminal extracellular domain contains motifs serving as docking sites for various ligands—primarily hyaluronan (HA), but also extracellular matrix (ECM) glycoproteins and proteoglycans, growth factors, cytokines and matrix metalloproteinases (Figure 1; Yu and Stamenkovic, 1999; Ponta et al., 2003). The aminoterminal domain of CD44 is separated from the plasma membrane by a short stem structure with proteolytic cleavage sites for such metalloproteinases as disintegrin, metalloproteinase domain-containing proteins 10 and 17 (ADAM-10 and ADAM-17) or membrane type 1-matrix metalloproteinase (MT1–MMP; Okamoto et al., 1999; Nagano and Saya, 2004). The cleavage of CD44 triggers the release of cells bound to HA—a process important in the regulation of cell migration. Notably, the stem region is the variable portion of the protein due to the alternative splicing and insertion of exons 6–15. The C-terminal cytoplasmic tail region plays a crucial role in the CD44 involvement in intracellular signal transduction. It has been shown that it binds to cytoskeletal elements such as ankyrin, the ERM proteins: ezrin, radixin, and moesin, and moesin-ezrin-radixin-like protein (MERLIN; Martin et al., 2003), it also interacts with signaling molecules, namely members of Src family kinases (SFKs) Src, Lck, Fyn and Lyn (Ponta et al., 2003), and activators of small Rho GTPases (Bourguignon et al., 2005). In addition, the cytoplasmic tail of CD44 may be cleaved off by γ-secretase and translocated to the cell nucleus, where it acts as a transcriptional regulator (Nagano and Saya, 2004).

The human gene for CD44 consists of 20 exons and is located in the 11p13 locus (Thorne et al., 2004). The most common form of CD44, also called standard CD44 (CD44s), is encoded by exons 1–5, 16–18 and 20. There also exists a large number of CD44 variants (CD44v), generated by extensive alternative splicing and containing the “standard” exons with a combination of exons 6–15 (Ponta et al., 2003). The predominant CD44 isoform expressed in the nervous system is the standard form (Sretavan et al., 1994; Jones et al., 2000; Bouvier-Labit et al., 2002), although the presence of CD44 splice variants has been demonstrated in normal human brain tissue (Kaaijk et al., 1997), as well as in certain primary tumours of the brain and peripheral nerve (Kaaijk et al., 1995; Sherman et al., 1997; Resnick et al., 1999).

## Expression and Activation in the Nervous System

Originally, CD44 expression was reported to take place predominantly in the white matter of the human brain (McKenzie et al., 1982; Bignami and Dahl, 1986; Cruz et al., 1986). Likewise, further studies have shown that the source of CD44 protein was glial cells, in particular astrocytes (Girgrah et al., 1991; Vogel et al., 1992). *In vitro* studies with the use of primary cell cultures from human foetal and adult brain have evidenced the expression of CD44 in astrocytes and oligodendrocytes (Moretto et al., 1993; Bouvier-Labit et al., 2002). The subsequent analysis of developing brain has identified CD44 as a marker for astrocyte-restricted precursor cells (ARPs) committed to give rise to astrocytes *in vitro* and *in vivo*, in both rodent and human tissue (Liu et al., 2004). In mouse cerebellum, CD44 expression was observed not only in astrocyte precursor cells, but also in neural stem cells and oligodendrocyte precursor cells (OPCs) at early postnatal stages (Naruse et al., 2013). CD44 expression in glial cells has been further demonstrated by studies examining the role of this molecule in the Schwann cells in developing nerves (Sherman et al., 2000) and at the neuromuscular junction of the adult rat skeletal muscle (Gorlewicz et al., 2009). The immunostaining of surgical specimens of temporal cortex and hippocampus for CD44 revealed astrocyte diversity in the human brain in terms of CD44 expression (Sosunov et al., 2014). High expression of CD44 has been observed in astrocytes with long, unbranched processes and “fibrous”-like astrocytes, whereas the “protoplasmic” astrocytes, which display a “bushy” morphology, exhibit no CD44 expression. Likewise, during development, CD44 expression was limited specifically to Bergmann glia and fibrous astrocytes among three types of astrocytes in cerebellum (Naruse et al., 2013). Interestingly, the expression of CD44 in astrocytes was shut off during postnatal development of the cerebellum.

Although the number of reports point to glial expression of CD44 in the nervous system, and describe adult neurons generally as CD44-negative cells (Vogel et al., 1992; Akiyama et al., 1993; Jones et al., 2000), the neuronal expression has also been observed. Expression of different splice variants of CD44, namely CD44v4, CD44v5 and CD44v10, in neurons (axonal membranes and cytoplasm) of the cerebral cortex, putamen, thalamus, hippocampus, cerebellum and spinal cord, in addition to the expression of CD44s in the white matter astrocytes, has been found in human brain sections (Kaaijk et al., 1997). It has also been shown that the embryonic optic chiasm neurons expressed the standard isoform of CD44 protein (Sretavan et al., 1994), and that strong upregulation of CD44 took place in axotomised facial motoneurons, but not in the gray matter astrocytes (Jones et al., 1997). In yet another study, the cellular location of CD44 in the brain tissue in seven different brain lesion models has been investigated (Jones et al., 2000). The results demonstrated an inducible expression of CD44 in cholinergic neurons of the forebrain, brainstem and spinal cord post distant axotomy, as opposed to its upregulation in astrocytes and other non-neuronal cell types following a more severe trauma involving local disruption of the blood-brain barrier. The antigen has been found in the cell bodies, dendrites and axons of neurons. CD44 immunoreactivity has also been detected in the dentate gyrus inner molecular layer (IML) of the mouse hippocampus after pilocarpine-induced status epilepticus (SE), where it coincided with early mossy fiber sprouting (MFS; Borges et al., 2004). Recently, CD44 mRNA has been detected in the neurons of striatum, extended amygdala and certain hypothalamic, cortical and hippocampal regions of non-stimulated brain (Glezer et al., 2009), as well as in the central respiratory control system (ventral respiratory group—VRG) within the brain stem (Matzke et al., 2007). CD44 expression was also detected in developing cerebellar Purkinje and granule neurons, but was limited to granule neurons in the adult cerebellum (Naruse et al., 2013). The expression of CD44 protein in pyramidal neurons of rat hippocampus and cortex is also developmentally regulated (Skupien et al., 2014). It increases postnatally, peaks around day P10 and then decreases. Notably, electron microscopy analysis revealed the dendritic localization of CD44 immunostaining within the pyramidal neurons of the developing hippocampus. There can be several causes for the apparent discrepancies among the studies describing expression of CD44 in neurons and glia. First, the CD44 expression appears to strongly depend on the developmental stage, especially in neurons. In fact, the studies claiming neurons to be CD44-negative were done mainly in the adult brain (Akiyama et al., 1993; Jones et al., 2000). The discrepancies may result also form differences in sensitivity of detection methods, and the differences in CD44 expression levels in glia (higher expression) and neurons (lower expression). In addition, since CD44 is a subject to extracellular and intramembrane proteolysis, the antibodies against its N-terminus may fail to detect the molecule in cells in which the rate of proteolytic processing is high. Such differences in the CD44 proteolysis, however, remain to be investigated.

## Biological Function in the Nervous System

**Figure 2:**
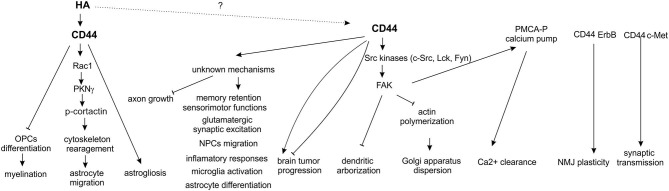
**Functions of CD44 in the nervous system**.

The main function ascribed to CD44 is acting as a receptor for HA—the key component of ECM in the brain playing a crucial role in many physiological and pathological processes, such as the neuronal development, synaptic plasticity (e.g., learning and memory), epileptogenesis, response to injury, neurodegeneration and brain tumour invasion (Kochlamazashvili et al., 2010; Wlodarczyk et al., 2011). The neuronal function of hyaluronic acid receptors, particularly CD44, in the nervous system remains to be elucidated, although there exists evidence that, among other processes, CD44 might be involved in the axon guidance during neuronal development (Figure 2). *In vitro* studies indicated that CD44 had an inhibitory effect on embryonic retinal axon growth (Sretavan et al., 1994), while experiments using specific anti-CD44 blocking antibodies demonstrated that the functional CD44 molecule in chiasmatic neurons was essential for axon crossing and axon divergence at the mouse optic chiasm (Lin and Chan, 2003). Furthermore, it appears that CD44 participates in the extension of axons from retinal ganglion cells growing on a laminin substrate (Ries et al., 2007).

Recently, a novel signaling pathway has been described in pyramidal neurons of the hippocampus and cerebral cortex, involving CD44 and Src tyrosine kinase-induced cascade, regulating Golgi apparatus morphology and dendritic tree arborization (Skupien et al., 2014). It was shown that the loss of CD44 resulted in an increase in the complexity of dendritic arbors in hippocampal neurons cultured *in vitro*, and in cortical neurons electroporated *in vivo*. Moreover, the knockdown of CD44 resulted in structural alteration of the Golgi apparatus, an organelle that is essential for mediating dendritic polarity, growth, and maintenance. Additionally, CD44 interacted with Src kinase in the brain, and the activation of both c-Src and its main substrate, focal adhesion kinase (FAK), was decreased upon CD44 knockdown.

The similar signaling pathway, involving CD44 and plasma membrane Ca^2+^ ATPase (PMCA), has been described in sensory neurons (Ghosh et al., 2011). The proposed molecular mechanism envisages PMCA as a point of cross-talk enabling the interaction of ECM and CD44, which regulates the Ca^2+^ signaling through the activation of SFKs (Src family kinases: Lck, Fyn) and FAK cascade. Since Ca^2+^ clearance from the neuronal cytoplasm regulates a number of Ca^2+^-dependent processes in neurons, including excitability, plasticity and neurotransmitter release, it appears that CD44 might be involved in all the above processes. Moreover, hyaluronic acid has been implicated in hippocampal synaptic plasticity by modulating postsynaptic L-type voltage-dependent Ca^2+^ channels (L-VDCCs; Kochlamazashvili et al., 2010). On the other hand, it has been demonstrated that activity of neuronal L-VDCCs is regulated by phosphorylation of its α_1c_ subunit by Src kinase (Bence-Hanulec et al., 2000; Gui et al., 2006). It would be interesting to investigate whether regulation of Src activity by CD44 can be involved in L-VDCCs-dependent synaptic plasticity.

In the nervous system and other tissues, CD44 can act as a co-receptor for receptor tyrosine kinases (RTKs) and serve as a platform for signaling molecule assembling. For instance, in the peripheral nervous system, CD44 enhances the neuregulin signaling by mediating the ErbB receptor heterodimerization in Schwann cells of the developing peripheral nerves (Sherman et al., 2000). In the adult rat nerve-muscle synapse, CD44 present in terminal Schwann cells has been shown to bind to ErbB3 receptors and to be involved in the neuromuscular junction plasticity (Gorlewicz et al., 2009). Moreover, the CD44v6 isoform can act as a co-receptor for c-Met *in vitro* (Orian-Rousseau et al., 2002). The CD44-c-Met interaction during embryogenesis has been further demonstrated by *in vivo* studies (Matzke et al., 2007) showing that c-Met is haploinsufficient in the CD44^−/−^ background. The CD44^−/−^ c-Met^+/–^ mice die at birth due to a breathing defect caused by impaired synaptic transmission in the respiratory rhythm generating network, and alterations in the phrenic nerve, while c-Met ^−/−^CD44^+/+^, and CD44^−/−^ c-Met^+/+^ mice develop normally and do not exhibit phenotypic abnormalities. The above results suggest that CD44 and c-Met are involved in synaptogenesis and axon myelination in the central and peripheral nervous systems.

It has been shown that CD44-deficient mice were born at the Mendelian ratio without any obvious developmental or neurological defects (Schmits et al., 1997). However, recently, Raber et al, applied a battery of behavioral and cognitive tests to determine if CD44-null mice display cognitive or other neurological disturbances (Raber et al., 2014). The results support an important role for CD44 in locomotor and sensorimotor functions, and in spatial memory retention, but do not specify whether these effects are due to the lack of CD44 in neurons or in glia. Given that CD44 is expressed in neural stem cells implicated in spatial memory (Oishi and Ito-Dufros, 2006; Deng et al., 2009; Naruse et al., 2013) the observed deficits can indicate that CD44 depletion in mice influences adult neurogenesis which in turn affects memory.

Interestingly, Matzke et al. (2007) have shown that the glutamatergic synaptic excitation in CD44 knockout mice was strongly reduced, while no change was detected in the glycinergic and GABAergic synaptic inhibition or in the overall synaptic activity of pre-Bötzinger complex neurons within the brain stem (Matzke et al., 2007). The above observations seem to indicate a potential role of CD44 in synaptic transmission, yet this requires more in-depth research. The absence of overt neurological defects in CD44 knockout mice might be explained by efficient compensation by another protein, all the more so since HA-mediated motility receptor (RHAMM) and intercellular adhesion molecule-1 (ICAM-1) have previously been shown to take over the CD44 role in the CD44 knockout mice (Nedvetzki et al., 2004; Olaku et al., 2011).

Although, the expression of CD44 in astrocytes has been described, the function of the interaction of HA and its receptor in these cells is poorly understood. It has been shown that interaction of hyaluronic acid with CD44 induce Rac 1-dependent PKNγ (protein kinase N-γ) activity which, in turn up-regulates the phosphorylation of the cytoskeletal protein, cortactin. This HA/CD44 interaction with Rac1-PKNγ leads to cytoskeleton activation and enhanced astrocyte migration (Bourguignon et al., 2007). Similarly, in neural precursor cells, overexpression of CD44 protein significantly enhanced the *in vitro* and *in vivo* trans-endothelial migration of these cells (Deboux et al., 2013). Moreover, it was shown that CD44 overexpression in glial precursor cells inhibits differentiation towards oligodendrocytes and increases the differentiation into astrocytes (Liu et al., 2004). Accordingly, a high molecular weight form of HA was also shown to inhibit maturation of OPCs into myelin-forming cells (Back et al., 2005).

## Association with the Nervous System Pathologies

The limited data from descriptive studies, indicating a widespread upregulation of CD44 expression in neurons and non-neuronal cells post brain injury, suggest an important role of this cell surface glycoprotein in the neuronal, glial and leukocyte response to trauma and in the nervous system repair (Jones et al., 2000; Shin et al., 2005). However, the molecular mechanisms underlying the above processes remain to be elucidated.

Few studies conducted to investigate the CD44 role in epileptogenesis have provided inconsistent results. It has been observed that CD44 was strongly upregulated in the dentate IML 3 days post pilocarpine-induced SE in mice, then it declined over the next 4 weeks (Borges et al., 2004). CD44 appeared to be one of the earliest proteins upregulated in the IML, which coincided with early MFS. On the other hand, repeated kainate injections did not induce any changes in the CD44 expression in the IML, which also correlated with MFS absence in the mice hippocampus. In view of the above, it has been hypothesized that CD44 was involved in the response to axon terminal degeneration and/or neuronal reorganization preceding MFS. Contrary to the above observations, studies on CD44 expression in an *in vitro* model of MFS have shown that high CD44 expression in the molecular layer coincided with minimal MFS, and that reduced CD44 expression/function following the kainic acid (KA) treatment or use of blocking antibodies was associated with increased MFS (Bausch, 2006). The time course of KA-induced decrease in CD44 expression corresponded with the temporal progression of KA-induced MFS in hippocampal slice cultures, suggesting that reduced CD44 expression might contribute to MFS. HA has been also implicated in epileptogenesis. Studies with the use of microelectrode array recording and Ca^2+^ imaging in hippocampal neurons cultured *in vitro* revealed that enzymatic removal of HA by hyaluronidase induced epileptiform activity in neuronal network (Vedunova et al., 2013). Additionally, mice deficient in HA synthase (HAS) genes, especially Has 3 knock-outs, exhibit epileptic phenotype along with a pronounced reduction in the level of tissue HA (Arranz et al., 2014).

Recently, it was shown that the silencing of CD44 expression during the early development of neuronal cells exerted a significant protective effect on young neurons that were exposed to subtoxic conditions, by preventing dendritic shortening induced by glutamate exposure (Skupien et al., 2014). Therefore, CD44 might be a novel therapeutic target in neurological disorders in which alterations in dendritic tree arborization have been observed.

One of a few studies with the use of CD44 knockout mice has provided evidence for the potential role of CD44 in the response to ischaemia in brain tissue (Wang et al., 2002). These findings indicated that CD44 deficiency in mice protected their brain from cerebral ischemia injury, and it has been suggested that this effect might be associated with selective reduction in inflammatory cytokines, for instance interleukin-1b. These results are further supported by studies showing that CD44 expression in activated microglia can be involved in the pathogenesis of neuroinflamatory diseases. Up-regulation of CD44 expression in microglia/macrophages were observed after transient or permanent forebrain ischemia in rats (Wang et al., 2001; Kang et al., 2008), in experimental cryolesions, a model for rat brain injury (Shin et al., 2005) and in mouse model of amyotrophic lateral sclerosis (ALS; Matsumoto et al., 2012).

Increased expression of CD44 and HA accumulation has also been observed in the brain white matter of patients with multiple sclerosis (MS), as compared with the normal brain tissue (Girgrah et al., 1991; Back et al., 2005). Consistently, elevated levels of both CD44 and HA has been detected in areas where there was loss of myelin, in the acute and chronic lesions in mice with experimental autoimmune encephalomyelitis (EAE), a murine model of MS (Back et al., 2005). Transgenic mice that overexpressed CD44 under the control of a myelin-specific promoter had widespread CNS dysmyelination and progressive demyelination that occurred in the absence of an inflammatory response (Tuohy et al., 2004). These findings provide strong evidence that CD44 proteins expressed by oligodendrocytes and Schwann cells, play a role in promoting demyelination. Moreover, HA staining intensity correlated with the levels of CD44 in CD44-overexpressing mice, indicating that HA accumulates in demyelinating CNS lesions as a result, at least in part, of elevated CD44 expression by glial cells (Back et al., 2005). HA inhibits remyelination and OPCs maturation after chemical demyelination of mouse white matter (Back et al., 2005) but, *in vitro* studies showed that blocked maturation of oligodendrocytes depends on HA interaction with Toll-like receptor 2 (TLR2) rather than with CD44 expressed by OPCs (Sloane et al., 2010). CD44 expression was strongly induced by activated astrocytes surrounding the demyelinated lesions suggesting that CD44 may play a role in facilitating inflammatory responses during reactive gliosis (Girgrah et al., 1991; Haegel et al., 1993). In MS the interactions between astrocytes and lymphocytes occurs during the entry of activated lymphocytes into the CNS, and in the sites of lesions. It was shown that CD44 is involved in direct contacts between T-cells and astrocytes in EAE mice (Haegel et al., 1993). Recently, an increased EAE disease severity and inflammation was demonstrated in CD44-KO mice (Flynn et al., 2013). CD44-deficient mice with EAE had more pro-inflammatory T-cell profile and increased permeability of the brain-blood barrier (BBB) than WT controls. The data suggest that CD44 acts as a negative regulator of inflammation with roles in T-cell differentiation, adhesion and trans-endothelial migration, and BBB permeability.

Protoplasmic astrocytes in patients with Alexander disease, a primary disorder of astrocytes, caused by heterozygous mutations in GFAP (glial fibrillary acidic protein), convert to reactive cells that lost their bushy-like morphology and become multinucleated and hypertrophic (Sosunov et al., 2013). This phenotypic conversion is accompanied by acquiring of CD44 by normally CD44-negative protoplasmic astrocytes of gray matter suggesting that CD44 can play a role in this process e.g., by regulation of changes in astrocyte shape. White matter of patients with vanishing white matter disease, that is associated with maturation defect of astrocytes and oligodendrocytes, is enriched in CD44-expressing astrocyte precursor cells and accumulates HA (Bugiani et al., 2013). Accumulation of HA synthesized by CD44-positive reactive astrocytes was also observed during chronic human neonatal white matter injury (Buser et al., 2012; Back and Rosenberg, 2014).

Chronically elevated CD44 expression and HA accumulation occur in non-human primate CNS with normal aging as a result of age-related astrogliosis and are linked to the aberrant accumulation of OPCs (Cargill et al., 2012). CD44 expression is also highly and persistently upregulated by astrocytes in brains of patients with Alzheimer’s disease (AD; Akiyama et al., 1993).

Several studies have demonstrated the correlation between high CD44 expression and poor prognosis in patients with brain tumour. CD44s and CD44v are frequently expressed in primary brain tumours and seem to be essential for the invasive growth of various CNS-derived tumour cell types *in vitro* and *in vivo* (Kuppner et al., 1992; Nagasaka et al., 1995; Sherman et al., 1997; Breyer et al., 2000; Monaghan et al., 2000; Pusch et al., 2010). The precise mechanism of CD44 action in the development and invasiveness of nervous system tumours has not been elucidated to date. In glioblastoma multiforme (GBM), the most aggressive brain tumour, CD44 inhibits the activation of the mammalian equivalent of Hippo signaling pathway and plays a key role in regulating the stress and apoptotic responses of human GBM cells (Xu et al., 2010). In addition, it has been found that a subset of human GBM cases showed high expression of CD44 in brain tumour stem-like cells (BTSC), and that the growth of these tumours might depend on CD44v6/AKT signaling pathway (Jijiwa et al., 2011). Su and colleagues have demonstrated that CD44 overexpression was induced by the Src kinase activity in malignant peripheral nerve sheath tumour (MPNST) cells, and that it contributed to tumour invasiveness (Su et al., 2003). By contrast, in neuroblastomas, CD44 expression is often low in advanced tumours (Combaret et al., 1997; Gross et al., 1997, 2000; Kramer et al., 1997). Shtivelman and Bishop (1991) have shown that several upstream *cis*-acting elements contribute to the downregulation of CD44 gene expression in neuroblastoma cells (Shtivelman and Bishop, 1991).

Overall, it appears that both the overexpression and lack of expression of CD44 might be involved in the development and invasiveness of various brain tumour types. The results of the performed studies are consistent with the hypothesis put forward by Herrlich and colleagues, stating that CD44 might play a dual role in cancer pathogenesis, acting as either an oncogene or a tumour-suppressing factor (Herrlich et al., 2000). By binding to growth factors and presenting them to their receptors, CD44 might be involved in the activation of tumour-promoting signaling pathways. On the other hand, in certain conditions, binding of HA might lead to the recruitment of tumour suppressor proteins (e.g., merlin) into the cytoplasmic tail of CD44 and result in cell growth arrest. Clearly, the posed hypothesis is elegant in its attempt to explain the dual role of CD44 in tumorigenesis. Nevertheless, its confirmation requires further research, for instance the identification of individual signaling cascades involved in the above-suggested mechanisms—particularly in the brain tissue.

In conclusion, there is growing evidence that the previously underappreciated CD44 molecule might be a key signal transducer in neurons, where it can act as a c-Met co-receptor to regulate synaptic transmission or through the activation of Src-dependent signaling pathways to influence dendritic arborization and calcium ions clearance. Additionally, CD44 can also modulate many other processes important for neuronal functions summarized in Figure 2, i.e., memory retention and axonal growth, but the exact cellular mechanisms of CD44 action in these phenomena are not discovered yet. One can expect that, at least some of the neuronal functions exerted by CD44, depend on HA binding but still the experimental evidences consistent with this notion are missing. In glial cells, unlike in neurons, binding of HA to CD44 receptor was shown to play an important roles in both physiological (myelination and oligodendrocyte maturation) and pathological (astrogliosis, demyelination) processes. However, except of the well-defined signaling cascade that include CD44/HA-dependent activation of small RhoGTPase Rac1 and PKNγ and subsequent cytoskeletal rearrangement in migrating astrocytes, CD44-related cellular mechanisms in glial cells (astrogliosis, microglia activation, oligodendrocyte and astrocyte maturation, see Figure 2) still remain to be elucidated. Furthermore, explanation of precise molecular mechanisms of CD44 action might potentially form basis for novel therapeutic interventions in a number of CNS disorders.

## Conflict of Interest Statement

The authors declare that the research was conducted in the absence of any commercial or financial relationships that could be construed as a potential conflict of interest.
